# Genetic control of temperament traits across species: association of autism spectrum disorder risk genes with cattle temperament

**DOI:** 10.1186/s12711-020-00569-z

**Published:** 2020-08-26

**Authors:** Roy Costilla, Kathryn E. Kemper, Enda M. Byrne, Laercio R. Porto-Neto, Roberto Carvalheiro, Deirdre C. Purfield, Jennifer L. Doyle, Donagh P. Berry, Stephen S. Moore, Naomi R. Wray, Ben J. Hayes

**Affiliations:** 1 Queensland Alliance for Agriculture and Food Innovation, The University of Queensland, Brisbane, Australia; 2 Institute for Molecular Bioscience, The University of Queensland, Brisbane, Australia; 3 Queensland Brain Institute, The University of Queensland, Brisbane, Australia; 4 Commonwealth Scientific and Industrial Research Organization (CSIRO), Agriculture and Food, Brisbane, Australia; 5 School of Agricultural and Veterinarian Sciences, Sao Paulo State University, Sao Paolo, Brazil; 6 Cork Institute of Technology, Bishopstown, Co. Cork Ireland; 7 Teagasc, Animal and Grassland Research and Innovation Centre, Moorepark, Fermoy, Co. Cork Ireland

## Abstract

**Background:**

Temperament traits are of high importance across species. In humans, temperament or personality traits correlate with psychological traits and psychiatric disorders. In cattle, they impact animal welfare, product quality and human safety, and are therefore of direct commercial importance. We hypothesized that genetic factors that contribute to variation in temperament among individuals within a species will be shared between humans and cattle. Using imputed whole-genome sequence data from 9223 beef cattle from three cohorts, a series of genome-wide association studies was undertaken on cattle flight time, a temperament phenotype measured as the time taken for an animal to cover a short-fixed distance after release from an enclosure. We also investigated the association of cattle temperament with polymorphisms in bovine orthologs of risk genes for neuroticism, schizophrenia, autism spectrum disorders (ASD), and developmental delay disorders in humans.

**Results:**

Variants with the strongest associations were located in the bovine orthologous region that is involved in several behavioural and cognitive disorders in humans. These variants were also partially validated in independent cattle cohorts. Genes in these regions (*BARHL2*, *NDN*, *SNRPN*, *MAGEL2*, A*BCA12*, *KIFAP3*, *TOPAZ1*, *FZD3*, *UBE3A*, and *GABRA5*) were enriched for the GO term neuron migration and were differentially expressed in brain and pituitary tissues in humans. Moreover, variants within 100 kb of ASD susceptibility genes were associated with cattle temperament and explained 6.5% of the total additive genetic variance in the largest cattle cohort. The ASD genes with the most significant associations were *GABRB3* and *CUL3*. Using the same 100 kb window, a weak association was found with polymorphisms in schizophrenia risk genes and no association with polymorphisms in neuroticism and developmental delay disorders risk genes.

**Conclusions:**

Our analysis showed that genes identified in a meta-analysis of cattle temperament contribute to neuron development functions and are differentially expressed in human brain tissues. Furthermore, some ASD susceptibility genes are associated with cattle temperament. These findings provide evidence that genetic control of temperament might be shared between humans and cattle and highlight the potential for future analyses to leverage results between species.

## Background

Temperament traits are of high importance across species. In humans, temperament traits include personality and behavior phenotypes such as extraversion, openness and neuroticism, and are genetically correlated with several psychiatric disorders, both common (such as major depression and anxiety disorders) and less common (such as schizophrenia and autism) [1–6]. In cattle, and other livestock species, temperament traits are of welfare and commercial importance because more docile animals can grow faster, are easier to transport and feed, and can have superior meat quality [7–12]. Moreover, reactive animals can also endanger the safety of their contemporaries and also their human handlers.

Several studies on animal species have suggested that phenotypic and genetic overlap for temperament between humans and other mammalian species. A long-running genetic study of behavior in foxes found an overlap between genes that are involved in aggression, sociability and anxiety in this species and autism spectrum and bipolar disorders in humans [13]. In cattle, several risk genes for human behavior and psychiatric disorders are also associated with temperament, docility and aggressiveness [14–17]. In terms of phenotypes, many studies have documented that characteristics such as hypersensitivity to sensory stimuli, fear in novel situations, visual thinking/ability to recall detail, are shared between animals and autistic people [18–20].

Temperament in cattle is measured as the response of the animal to handling or forced movement by humans [21]. Various measures for cattle have been proposed, including an electronically recorded phenotype called flight time. Flight time is defined as the time taken for an animal to cover a short-fixed distance after release from an enclosure [7, 21]. Animal responses to this test have been shown to be repeatable over time [7], and thus, flight time is routinely used by the cattle industry to measure temperament. More generally, flight time belongs to a group of restraint tests that measure both the animal’s response to human proximity and physical restraint. Estimated heritabilities for cattle temperament are moderate (average of 0.36 with a range of 0.05 to 0.70) with the variation in reported estimates due, in part, to study designs, but also to breed differences [7, 14–16, 21].

In humans, temperament is interchangeable with personality [7, 22–25] and is generally measured by using one of two taxonomies: the five-factor model [26, 27] or Cloninger temperament scales [22, 23]. The “Big-five” model has five domains: Extraversion, Neuroticism, Agreeableness, Conscientiousness and Openness, while Cloninger’s model has four main dimensions: Novelty Seeking, Harm Avoidance, Reward Dependence and Persistence. An important point to note is that variation of temperament domains/dimensions is thought to be influenced by activity in specific neurotransmitter pathways [25]. Based on twin and family studies, heritabilities for personality traits in humans are estimated to be around 0.4 [28].

There is growing empirical evidence that suggests that orthologous genes control complex traits in different mammalian species [29–32]. For instance, Pryce et al. [29] showed that genes associated with height in humans are also associated with stature in cattle. In a recent comparison between humans, cattle and dogs, Bouwman et al. [30] found that the genetic architecture of stature in cattle is similar to that in humans, in that it is highly polygenic with many polymorphisms of small effects; multiple loci associated with stature were shared across the three-species investigated. A comprehensive review of known mutations in genes that affect body size in domestic species, mice and humans identified many common genes [31].

Here, our aim was to test the hypothesis that, as for stature, a common set of genes control temperament across two mammalian species, namely humans and cattle. We hypothesized that genetic loci that contribute to variation between individuals will be shared across these species, and hence we used humans as a model organism for cattle to investigate the effect of genes related with human behavior and psychiatric disorders in cattle temperament. In particular, we tested the enrichment of polymorphisms associated with cattle temperament in genes that are involved in four human psychiatric and personality disorders traits, which have been most comprehensively studied: neuroticism (NEU) [33], schizophrenia (SCZ) [34], autism spectrum disorder (ASD) [35], and developmental delay disorders related to brain and/or cognition (DDD) [36].

## Methods

### Phenotypes and genotypes

The phenotype used was flight time, which is the electronically recorded time taken for an animal to cover 1.7 m after being released from the weighing box. This temperament phenotype was measured for three tropically adapted beef cohorts: The Cooperative Research Centre-CRC dataset (Brahman and TropComp1) and a Tropical composite dataset 2 (TropComp2, with a different breed composition). The number of animals and other details of these cohorts can be found in (see Additional file 1: Table S1) and [15]. Prior to the analysis, we pre-processed phenotypes using a natural logarithm transformation and standardized it to have a mean of 0 and a variance of 1 within each cohort.

Animals in the cohorts were genotyped in commercial bovine arrays, either the Illumina BovineSNP50 or the Zoetis HD50K (50K markers). Monomorphic single nucleotide polymorphisms (SNPs) and those with more than 10% of animals with GenCall scores lower than 0.6 were excluded. Animals with GenCall scores lower than 0.9 were also removed. Following standard protocols for bovine data [30, 37–40], genotypes were imputed twice, first to SNPs on the Illumina Bovine HD (HD) array and then to whole-genome sequence (WGS). The former was performed in FImpute2 [41] using a reference panel of 1500 cattle of relevant breeds genotyped for the HD array. The latter was done using Eagle [42], Minimac3 [43] and a multi-breed reference panel of 472 sequenced animals, average of 11× coverage, from the 1000 Bull Genomes Project run 6 [37, 44]. The estimated imputation accuracy was reasonably high (average Minimac r^2^ = 0.88).

**Fig. 1:**
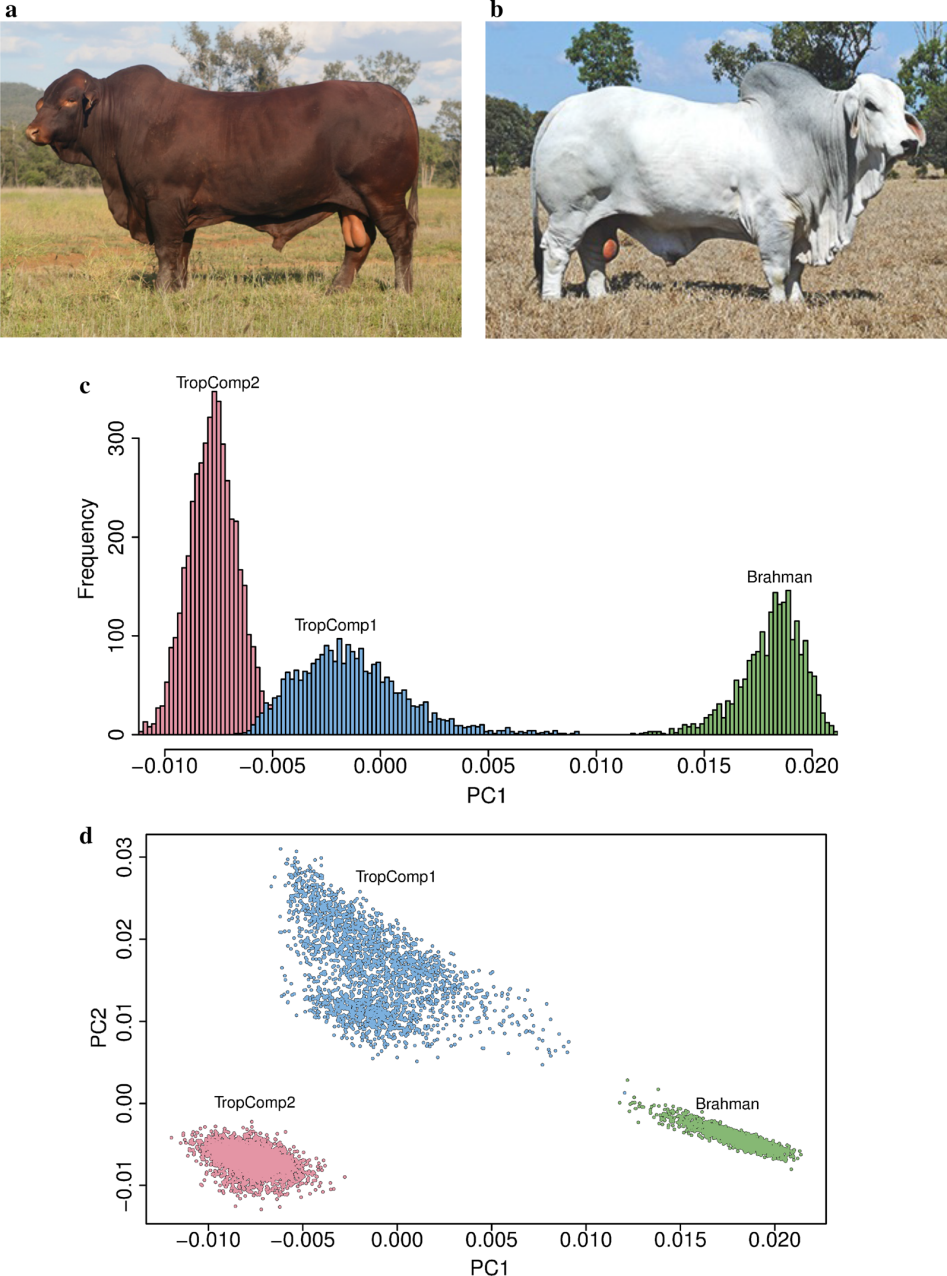
Cattle cohorts included in the meta-analysis. **a** Tropical composite (Santa Gertrudis) and **b** Brahman bulls. **c** Distribution of the first principal component (PC) for all three cohorts. The first PC is proportional to the indicine content (*Bos indicus*) of the animal with the Brahman population having a higher content than any of the composite breeds. **d** Joint distribution of first and second PC for all three cohorts

SNPs were then processed through a standard bovine quality control pipeline, filtered out within each cohort on minor allele frequency (MAF), which had to be lower than 10^−4^, extreme deviation from Hardy-Weinberg equilibrium (HWE), pHWE had to be lower than 10^−10^, and imputation quality (Minimac r^2^ < 0.6). MAF and HWE filters were implemented using PLINK1.9 [45]. Only autosomal SNPs were kept for further analysis. These filtering steps resulted in a similar number of imputed sequence variants as reported in other cattle studies [30, 37, 40]. Details of the genetic similarity (principal components (PC)) between the three cohorts are in Fig. 1. Due to the different levels of cross-breeding in the composite animals, even the first PC (proportional to indicine content [15]) could differentiate between the cohorts.

### Meta-analysis

We conducted genome-wide association studies (GWAS) on cattle flight time for three populations and conducted a meta-analysis of the results for a combined sample of 9223 animals and 28.4 million imputed biallelic variants, including both SNPs and small insertions and deletions (INDEL). The number of animals and variants varied slightly between cohorts, ranging from 2112 to 4586 animals and 24.8 to 28.2 million variants. Within each cohort, the model was fitted as:

1 $${\mathbf{y}} = {\mathbf{X \varvec{\upbeta}}} + {\mathbf{Zg}} + {\varvec{\upvarepsilon}},$$

where ${\mathbf{y}}$ is a vector of phenotypes (standardized natural logarithm of flight time), ${\varvec{\upbeta}}$ is a vector of fixed effects including the genotype for the candidate SNP and all covariates (mean, log(age), and contemporary group (year, stud, and sex)), ${\mathbf{g}}$ is a vector of total additive genetic effects with ${\mathbf{g}} \sim {\text{N}}\left( {0,{\mathbf{G}}{{\sigma }}_{{ {\text{g}}}}^{2} } \right)$, where ${\mathbf{G}}$ is the genomic relationship matrix (GRM) generated from imputed sequence variants, ${{\sigma }}_{{ {\text{g }}}}^{2}$ is the additive genetic variance, and ${\varvec{\upvarepsilon}}$ is a vector of random residuals ${\varvec{\upvarepsilon}}\sim {\text{N}}(0,{\mathbf{I}}{{\sigma }}_{{{\varepsilon }}}^{2}$). ${\mathbf{X}}$ and ${\mathbf{Z}}$ are design matrices for the fixed and random effects, respectively. The GRM was constructed following [46]. Note that this is an additive model for the SNP that assumes that the effect of having two copies of the non-reference allele is twice the effect of having one only.

GWAS were performed in GCTA [47]. Results for all cohorts were combined using a fixed effects inverse-variance weighted meta-analysis as implemented in METAL [48]. We used a significance threshold of P < 5$\times$10^−8^, which corresponded to a false discovery rate (FDR) of 0.01. Figures throughout the article were generated using *R* [49]. The meta-analysis lead variants were identified using clumping in PLINK1.9 [45] with parameters: P <5 $\times$10^−8^, 5-Mb windows, and r^2^ = 0.1.

### Validation test of variants identified in the meta-analysis

Statistically significant variants in the meta-analysis were tested for replication using GWAS summary statistics for docility scores in five independent cohorts of *Bos taurus* breeds (Angus, Charolais, Hereford, Limousin, and Simmental) from the Irish national breeding program [50]. Docility score is a temperament phenotype in cattle that reflects cattle response to human handling on an ordinal scale (1 = “Aggressive” to 10 = “Docile”) and is recorded by trained classifiers. On this scale, we assume docility score to be positively correlated with flight time. Variants identified in the meta-analysis with a P < 0.05 in the validation GWAS were considered validated. To approximate a null distribution for the number of variants expected by chance in the validation GWAS using this criterion, we also ran a permutation test (10,000 replicates per cohort, 50,000 in all five cohorts). The number of animals and variants for the validation cohorts and a sign test of concordance for the latter are in (see Additional file 1: Tables S2 and S3), respectively. A summary of the methodology for the GWAS in these validation cohorts is also available in Additional file 2.

### Mapping and functional annotation of genes from the meta-analysis

Two methods were used to annotate the variants that were identified in the meta-analysis to putative candidate genes. First, we identified protein-coding genes within 200 kb of the clumping window that contained the top associated variant. Second, we ran a gene set-based association analysis using summary statistics from the meta-analysis in fastBAT/GCTA [51] using the combined genotypes for the three populations as a linkage disequilibrium (LD) reference panel.

Functional annotation of human orthologous genes identified in the meta-analysis was carried out using STRINGv11 [52]. We also investigated differential expression (DE) of human orthologous genes identified in the meta-analysis in GTEx v6 [53] tissues using FUMA [54].

### Enrichment of NEU, SCZ, ASD and DDD genes in cattle temperament

We located bovine orthologous genes associated with NEU [33], SCZ [34], ASD [35], and DDD [36] using the UMD3.1 bovine reference genome. The orthology annotation was done as follows. Highly conserved genes were obtained using bovine orthologs genes from Biomart Ensembl 94 with the following quality control criteria for the genes: they had to be at least 60% identical to target human genes, to have a protein-coding function, and to be located on autosomal chromosomes. Given that the genes involved in developmental delay disorders are related to a very broad spectrum of disease/phenotypes with varying levels of evidence for functional consequence, DDD genes were further filtered to have a “confirmed” status, “loss of function” mutation consequence, and to be related to “Brain/Cognition” phenotypes.

SNPs and INDEL were mapped to genes based on boundaries of ±100 kb of the gene start and end sites. We chose this window size because of the levels of LD in the tropically adapted animals used in the analysis. A previous study used a window of 500 kb for *Bos taurus* breeds [29] which have longer tracts of LD when compared with *Bos indicus* and composite breeds [55]. In addition, [56] showed that even at 70 kb the levels of LD in tropically adapted beef cattle are not small (0.1 3 < r2 < 0.16). Thus, a 100-kb window provided an intermediate compromise. In total, we tested the effects for 263, 577, 101 and 63 bovine orthologous genes that are involved in SCZ, NEU, DDD, and ASD, respectively (see Additional file 1: Tables S4–S7). The total number of variants in these gene sets in our larger cohort (TropComp2) were 584,889, 382,996, 287,480 and 159,455 variants for SCZ, NEU, DDD, and ASD, respectively.

We tested for enrichment of SNP associations with bovine temperament in genomic regions surrounding bovine orthologous genes to the above four disorders in two ways: (1) using data from the association meta-analysis, we compared observed versus expected distribution of χ^2^ test statistics and identified SNPs that were associated with flight time (P < 1$\times$10^−4^), and (2) using data from the largest cohort (TropComp2 with 4586 animals), we estimated the percentage of additive genetic variance explained by these SNPs using a model with two GRM, one for variants around (100 kb) genes in each set and another one for the remaining ones in the bovine genome:

2 $${\mathbf{y}} = {\mathbf{X\varvec{\upbeta} }} + {\mathbf{Z}}_{1} {\mathbf{g}}_{1} + {\mathbf{Z}}_{2} {\mathbf{g}}_{2} + {\varvec{\upvarepsilon}},$$

$${\mathbf{g}}_{1} \sim {\text{N}}\left( {0,{\mathbf{G}}_{1} {{\sigma }}_{{ {\text{g}}1 }}^{2} } \right),$$

$${\mathbf{G}}_{1} = {\mathbf{W}}_{1} {\mathbf{W}}'_{1} /k_{1} ,$$

$$w_{1ij} = \frac{{x_{ij} - 2p_{j} }}{{\sqrt {2p_{j} \left( {1 - p_{j} } \right)} }} ,$$

$$x_{ij} = 0,1,2,\quad i = 1, \ldots ,n,\quad j = 1, \ldots ,k_{1} ;$$

$${\mathbf{g}}_{2} \sim {\text{N}}\left( {0,{\mathbf{G}}_{2} {{\sigma }}_{{ {\text{g}}2 }}^{2} } \right),$$

$${\mathbf{G}}_{2} = {\mathbf{W}}_{2} {\mathbf{W^{\prime}}}_{2} /k_{2} ,$$

$$w_{2ij} = \frac{{x_{ij} - 2p_{j} }}{{\sqrt {2p_{j} \left( {1 - p_{j} } \right)} }},$$

$$x_{ij} = 0,1,2,\quad i = 1, \ldots ,n,\quad j = 1, \ldots ,k_{2} ,$$

$$k_{1} + k_{2} = k.$$

In addition to the terms defined in Eq. (1), ${\mathbf{Z}}_{1}$ and ${\mathbf{g}}_{1}$ denote the design matrix and additive genetic effects for variants in the gene set tested ($k_{1}$). ${\mathbf{Z}}_{2} {\text{and }}{\mathbf{g}}_{2}$ represent the design matrix and additive genetic effects for variants in the rest of the bovine genome ($k_{2}$). ${\mathbf{G}}_{1}$, ${\mathbf{G}}_{2 }$ and ${\mathbf{W}}_{1}$, ${\mathbf{W}}_{2}$ are the corresponding GRM matrices and standardized genotype matrices for variants in these two gene sets. $p_{j}$ denotes the non-reference allele frequency for variant $j$. We estimated ${{\sigma }}_{{ {\text{g}}1 }}^{2}$ and ${{\sigma }}_{{ {\text{g}}2 }}^{2}$ from Model (2) using GCTA [47].

## Results

### Meta-analysis of bovine temperament

Imputed whole-genome sequence based GWAS of cattle flight time were conducted in three cohorts of tropically adapted breeds (one *Bos indicus* and two composite *Bos taurus*/*Bos indicus*) with 9223 animals and 28.4 million variants (Fig. 1). Genomic heritability estimates for cattle flight time in these cohorts were moderate to high, ranging from 0.26 (0.03) to 0.49 (0.05), and in close agreement with previous estimates for cattle temperament using just pedigree (ancestry) information [7, 15, 16, 21]. For each cohort, the ratio of the observed to expected median test statistic showed no evidence of genomic inflation (0.937 $\le {{\lambda }}$ gc $\le$ 1.001, [see Additional file 1: Table S1]).

**Fig. 2:**
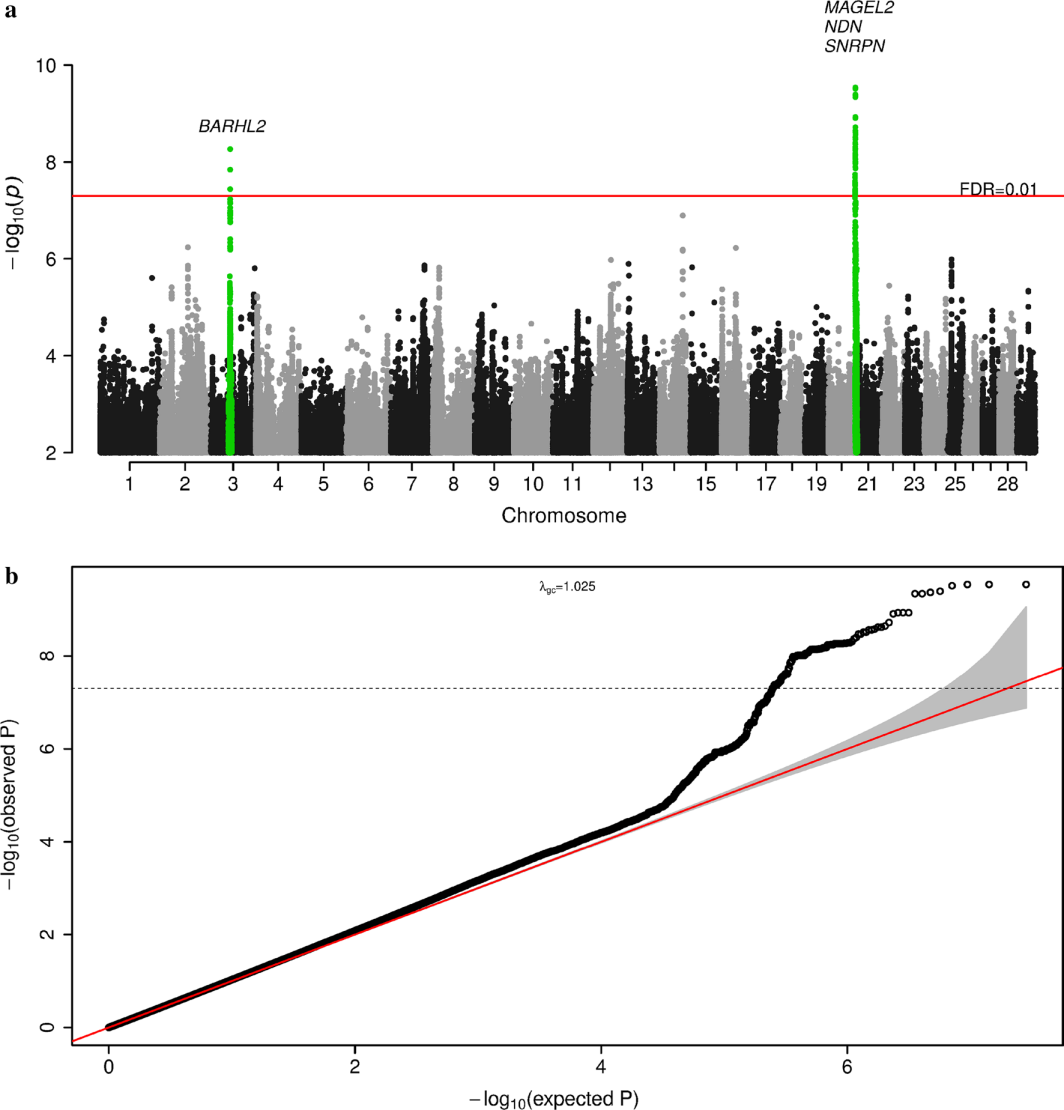
GWAS meta-analysis results for cattle temperament. Shown are association statistics [− log10(P)] ordered by genome position (**a**) and the corresponding QQ-plot (**b**). Candidate genes shown in (**a**) are protein-coding genes mapped by physical distance (within 200 kb of the clumping window that contained lead variant)

In the association meta-analysis (Fig. 2), we identified 115 genome-wide significant variants (P < 5$\times$10^−8^ and FDR = 0.01) which were mostly intergenic (108 intergenic, 5 downstream and 2 upstream of genes). These variants mapped to two independent genomic regions (clumping), located on bovine chromosomes 3 and 21. The regions contained four protein-coding genes *BARHL2*, *NDN*, *SNRPN*, and *MAGEL2* (Fig. 2 and Table 1). GWAS summary statistics for all variants in the meta-analysis are part of this manuscript and are provided in Additional file 3.

**Table 1:** Estimated lead variants (2) associated with cattle flight time in the meta-analysis

Chr	bp	A1/A2	Freq (A1)	b (A1)	P	%Vg^a^	Dir^b^	Variant type	Candidate genes^c^
3	52,892,109	A/C	0.56	− 0.089	2 × 10^−9^	1.50	–	Intergenic	*BARHL2* (196 kb)
21	1,058,688	A/C	0.48	− 0.107	3 × 10^−10^	2.19	–	Intergenic	*MAGEL2* (274 kb), *NDN* (321 kb) *SNRPN* (1033 kb)

Lead variants are obtained using clumping for all variants with P < 5 × 10^−8^, clumping windows of 5 Mb and r^2^ = 0.1

^a^%Vg = 2*Freq_A1*_Freq_A2*_b^2^/Vg*100, Vg = 0.259 (TropComp2)

^b^Direction of SNP effect in each cohort of the meta-analysis (TropComp1, Brahman, TropComp2)

^c^Protein-coding genes within 200 kb of the clumping window. Distance from top variant in parenthesis

Through gene set-based association analysis [51], we further identified six protein-coding genes that were significantly associated with flight time (*ABCA12, KIFAP3, TOPAZ1, FZD3, UBE3A,* and *GABRA5* [see Additional file 1: Table S8]). The variants identified in the meta-analysis of cattle flight time were thus mapped to 10 protein-coding genes [see Additional file 1: Table S9].

**Fig. 3:**
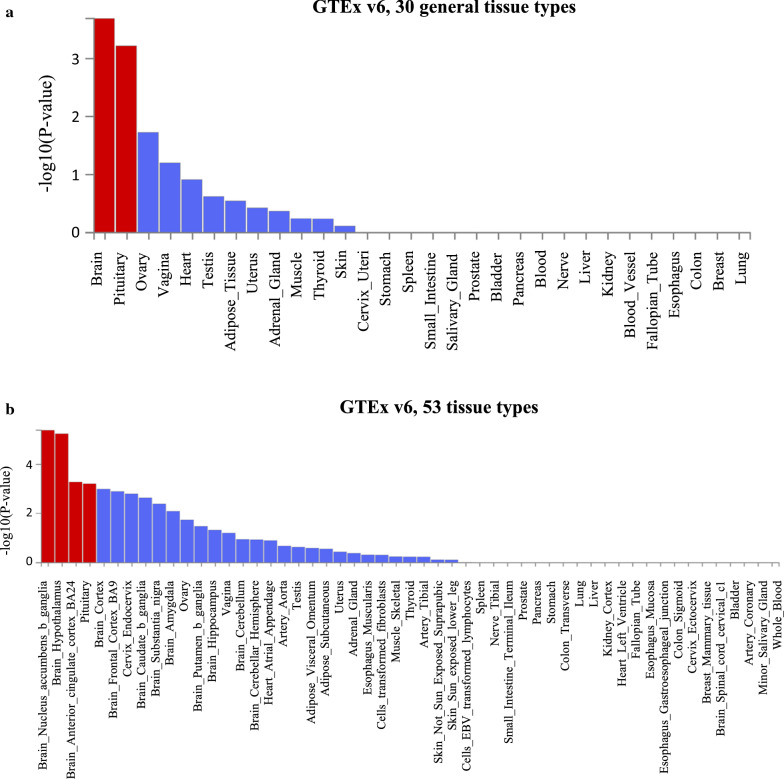
Differential expression (up-regulation) in GTEx tissues of human orthologous genes associated with cattle temperament in the meta-analysis. Tissues for which up-regulation is statistically significant after Bonferroni correction are shown in red. Ten protein-coding genes were associated with cattle temperament in the meta-analysis: *BARHL2*, *NDN*, *SNRPN*, *MAGEL2*, *ABCA12*, *KIFAP3*, *TOPAZ1*, *FZD3*, *UBE3A*, and *GABRA5*. Analysis were performed in FUMA

In terms of functional annotation of human orthologous genes, we found that three (*NDN*, *BARHL2*, and *FZD3*) out of 10 genes identified in the meta-analysis were enriched for the GO term neuron migration (GO term: 0001764, FDR = 0.01) and were also differentially expressed in several human tissues (Fig. 3). In particular, these genes were up-regulated in: 2/30 general tissue types (brain and pituitary) and 4/53 tissue types (brain nucleus accumbens basal ganglia, brain hypothalamus, brain anterior cingulate cortex, pituitary).

### Validation tests of variants in five independent cattle populations

Of the 115 variants from two genomic loci identified in the meta-analysis, 73 were also significantly associated with docility scores (P < 0.05 in the validation GWAS) in at least one of the replication cohorts. This number of validated variants is significantly larger than expected by chance (29.6 in 50,000 replicates, P < 2$\times$10^−5^). All validated variants were located on chromosome 21 and were mostly breed-specific (only 5 out of the 73 validated variants segregated in two breeds). When looking at the meta-analysis of identified variants within individual cohorts, we found that 30 out of 99 polymorphic variants were associated with docility scores in Charolais animals (P < 0.05 in the validation GWAS). The number of validated variants for Limousin and Simmental animals was 24 out of 99 and 24 out of 74, respectively. No variants were validated in the Angus and Hereford cohorts although 83 and 101 out of 115 were polymorphic. Further details for the variant validation and the permutation test can be found in (see Additional file 1: Table S2).

**Fig. 4:**
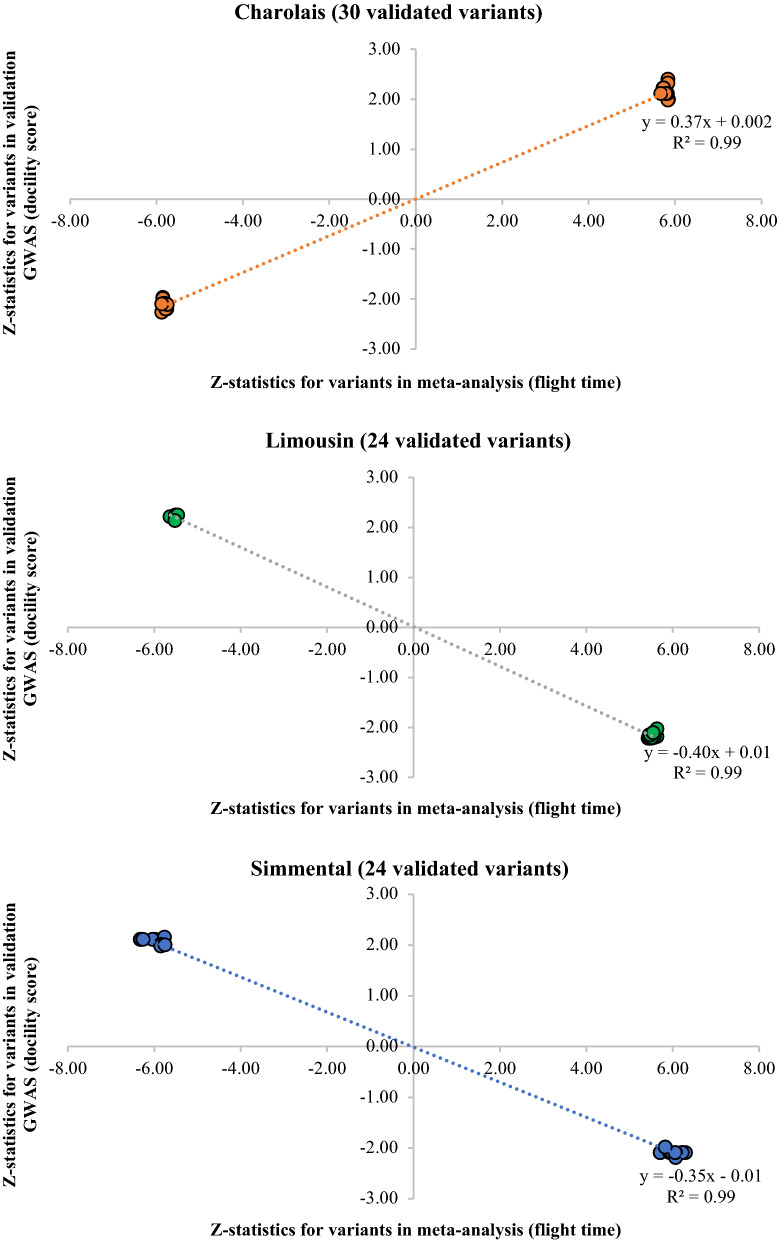
Validation of variants identified in the meta-analysis (115 variants) in five independent *Bos taurus* cohorts (P < 0.05 in validation cohort). Plots show Z-statistics for the variants in the meta-analysis (temperament) and validation GWAS (docility score). The allele substitution effect of variants associated with flight time is assumed to have the same direction as for docility scores (1 = “Aggressive” to 10 = “Docile”). Across all cohorts, there were 73/115 validated variants (P < 2 $\times$ 10^−5^, permutation test), all located on chromosome 21, and 30/73 variants had the expected direction (P = 0.01, sign test of concordance), all found in Charolais animals

All allele substitution effects for validated variants in the Charolais population had the same direction as in the meta-analysis but the opposite direction in the Limousin and Simmental populations, which implies that there are differences of variant effect direction across breeds (sign test of concordance, Fig. 4 and [see Additional file 1: Table S3]). This was the case for instance, for the top signal from the meta-analysis (SNP rs137773155 on chromosome 21, bp 1,058,688) which was validated in the Simmental population but with an allele substitution effect in the opposite direction to that estimated in the meta-analysis, e.g. a copy of the C allele at this SNP decreased the flight time in tropically adapted cattle but increased the docility score in the Simmental animals.

### Enrichment of NEU, SCZ, ASD and DDD genes in cattle temperament

**Fig. 5:**
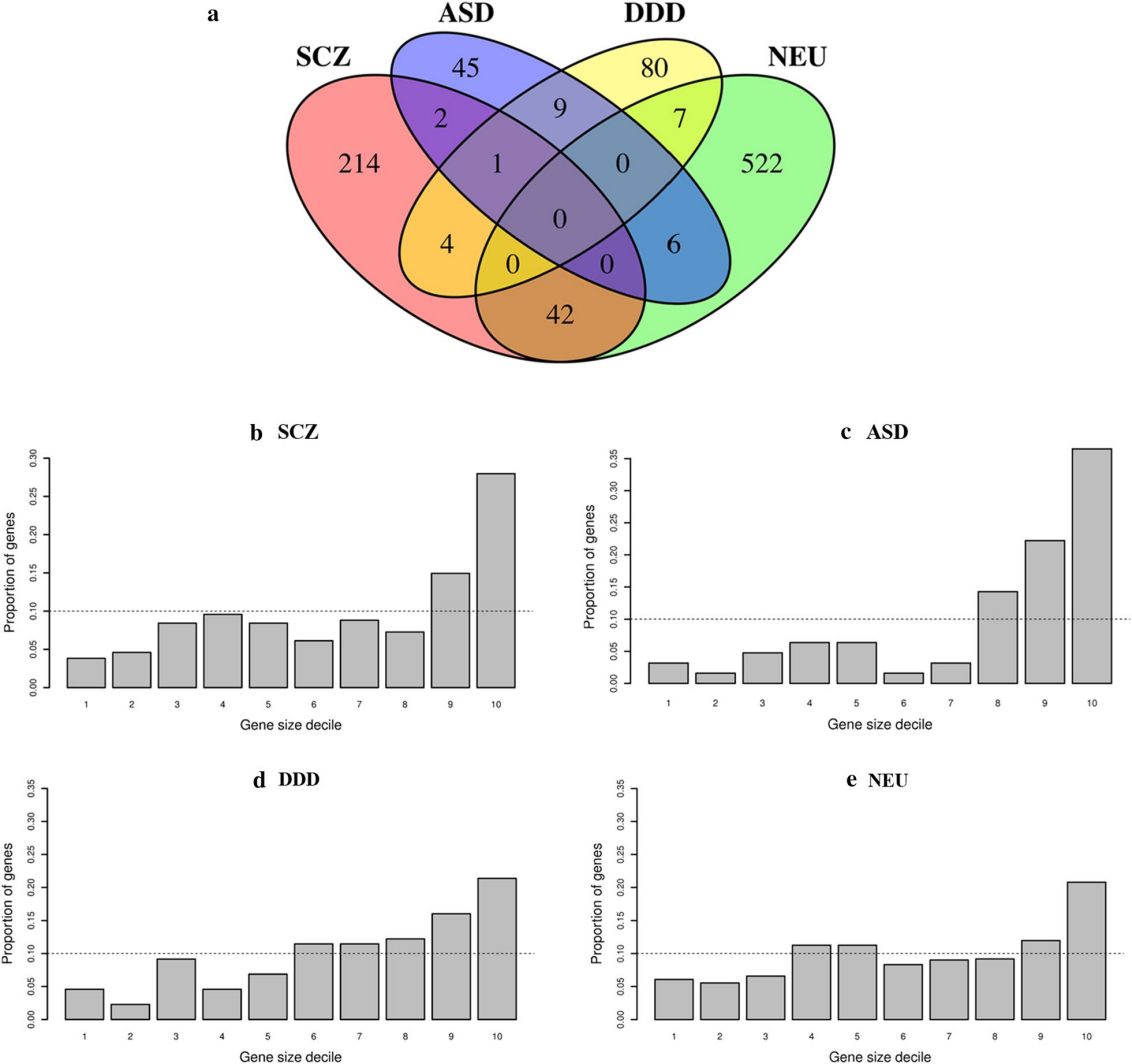
Bovine orthologous of genes involved in schizophrenia (SCZ), autism spectrum disorders (ASD), developmental delay disorders (DDD) and neuroticism (NEU). **a** shows the overlap between them. **b**–**e** show the decile distribution of the physical size of genes within each set. Horizontal dashed lines represent the expected proportion of genes on each decile

There were two salient characteristics of the bovine orthologous genes involved in the four disorders tested. First, there was very little overlap between the NEU, SCZ, ASD and DDD gene sets (Fig. 5, top panel). For instance, there was no single gene that was common among the four gene sets. Second, these gene sets tend to be very large in physical size (gene length) as is typical for brain-associated genes (Fig. 5, bottom panel).

We found no difference between the percentage of variance explained by variants located in or close (100 kb) to 577 NEU and 101 DDD susceptibility genes and variants in random gene sets of similar size. That is, the 450,283 and 332,396 variants that mapped to bovine orthologous to the NEU and DDD genes in the largest animal cohort (TropComp2) explained negligible variance, not different from zero, of the additive genetic variance in a model with one genomic relationship matrix (GRM) for these variants and another one with the remaining variants in the bovine genome (Eq. (2) in “Methods”). Results were similar for the other two bovine cohorts with no enrichment in bovine temperament of genes involved in NEU and DDD.

**Fig. 6:**
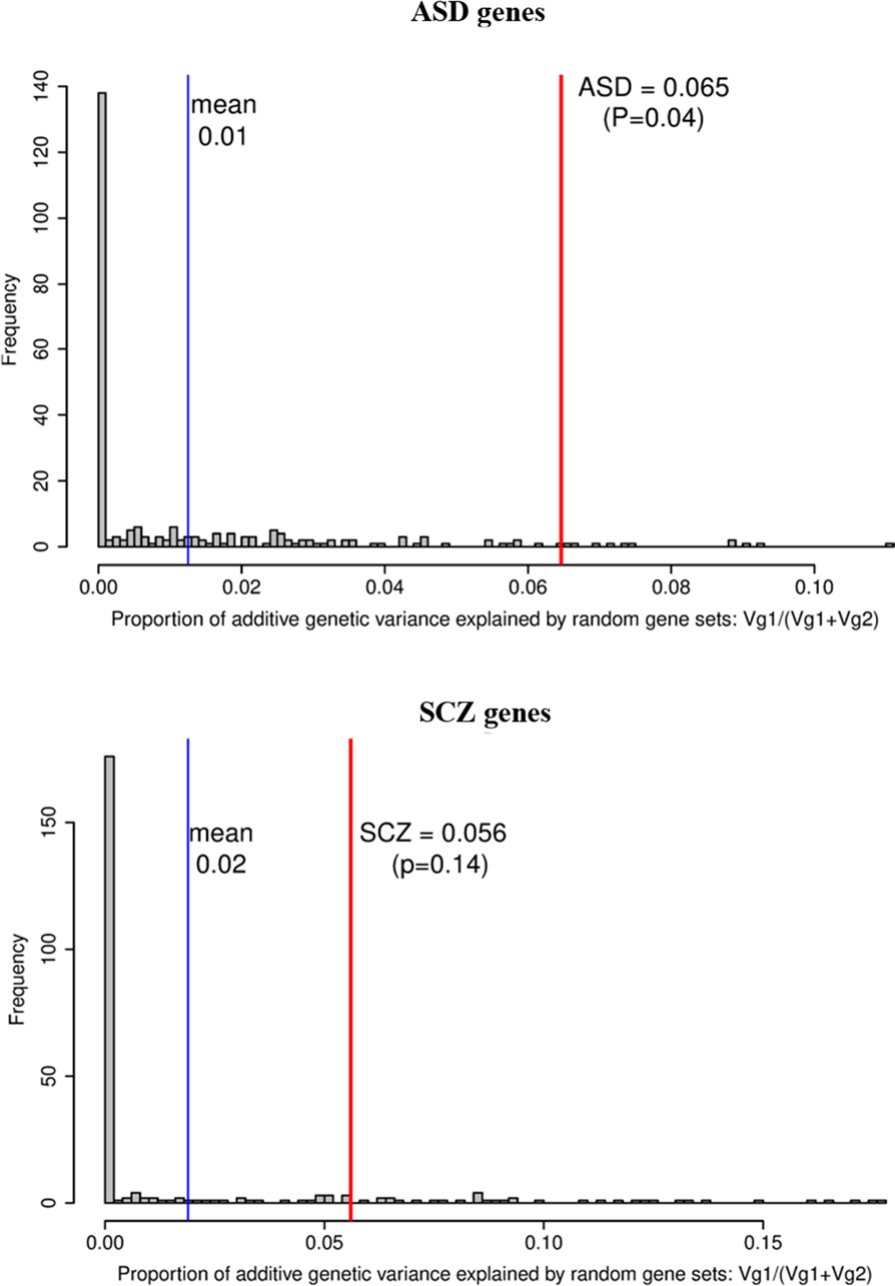
Randomized permutation test results over 250 replicates. Distribution of the proportion of genetic variance explained in the TropComp2 cohort by random gene sets for a model that fits simultaneously the variants within +/- 100 kb of the random gene set (Vg1) and the remaining variants (Vg2) in the bovine genome. Blue vertical lines display mean values of the distribution. Results for ASD (159,455 SNPs) and SCZ (584,889 SNPs) genes are also shown as vertical red lines

We found evidence of an association stronger than expected by chance between variants in the 63 ASD risk genes and cattle flight time. Out of the 183,880 SNPs in or close (100 kb) to bovine ASD orthologs in the meta-analysis, there were 13 SNPs associated with cattle flight time (P < 1×10^−4^) located in two regions on chromosomes 2 and 21 (see Additional file 4: Figure S1). Each of the two lead SNPs explained 0.9% of the additive genetic variance for cattle flight time (Table 2). The two candidate genes in these regions included bovine orthologs for the *gamma*-*aminobutyric acid type A receptor beta3 subunit* (*GABRB3*), and *cullin 3* (*CUL3*). Moreover, SNPs in and around the ASD genes explained a significantly higher percentage of the additive genetic variance in cattle temperament when compared to random gene sets of similar size in the largest cohort available (TropComp2). Variants in and around 63 bovine ASD orthologs explained 6.5% of the additive genetic variance in the model with two GRM (Eq. (2)) in the largest cattle cohort, while on average random gene sets of similar size (same number of genes and similar size of genes) as in the ASD set only explained 1% of the additive genetic variance (P = 0.04 over 250 random permutations, (Fig. 6)). Using data from a bovine gene expression atlas [57], we also confirmed that ASD genes are differentially expressed in bovine brain tissue [see Additional file 1: Table S10, Additional file 2, and Additional file 4: Figure S2].

**Table 2:** Estimated lead variants within ± 100 kb of ASD genes associated with cattle flight time in the meta-analysis

Chr	Bp	A1/A2	Freq (A1)	b (A1)	P	%Vg^a^	Dir^b^	Variant type	Candidate genes^c^
2	113,546,307	T/C	0.222	− 0.082	2.3 × 10^−5^	0.90	–	Intergenic	*CUL3* (59 kb)
21	4,115,188	A/G	0.344	− 0.072	8.0 × 10^−6^	0.90	-+-	Intergenic	*GABRB3* (28 kb)

Lead variants are obtained using clumping for all variants with P < 1 × 10^−4^, clumping windows of 5 Mb and r^2^ = 0.1

^a^%Vg = 2*Freq_A1*_Freq_A2*_b^2^/Vg*100, Vg = 0.259 (TropComp2)

^b^Direction of SNP effect in each cohort of the meta-analysis (TropComp1, Brahman, TropComp2)

^c^Protein-coding genes within 200 kb of the clumping window. Distance from top variant in parenthesis

Some evidence of an association stronger than expected by chance was also found for the 584,889 variants in or close to the 263 SCZ susceptibility genes in the largest cattle cohort. Combined together these variants explained 5.6% of the additive genetic variance in the model with two GRM (Eq. (2)). Although this result was not statistically significant (P = 0.14 over 250 replicates), random sets of genes of similar size to SCZ genes explained, on average, only 2% of the additive genetic variance (Fig. 6). The weak enrichment of SCZ genes was robust to an increasing number of permutations in the randomized permutation test (P = 0.13 over 1000 replicates). Moreover, the SCZ genes had very little overlap with the ASD genes (3 of 263 SCZ genes were also involved in ASD, Fig. 5).

## Discussion

Genome-wide association meta-analysis of three cattle cohorts measured for the temperament trait of flight time identified significant independent associations at two genomic regions on bovine chromosomes 3 and 21. Variants in these regions were validated in independent cattle cohorts and mapped to 10 human orthologous genes. These genes are biologically enriched in neuron migration and differentially expressed in brain and pituitary human tissues. The human genomic region (15q11-q13) that is orthologous to the top associated region in the flight time meta-analysis (start of bovine chromosome 21) encompasses genes that have been implicated in several behavioural and cognitive disorders, including childhood obesity [58], epilepsy [59] and the genetic disorders Angelman and Prader-Willi syndromes [60, 61]. Mouse and rat models have shown that copy number variation in this region is involved in feeding disorders, delayed motor skills development, and altered circadian rhythm [62–64]. In cattle, variants in this region, located at the start of chromosome 21, are associated with aggressiveness [14] and fertility [65, 66]. There is also growing evidence for the role of synaptic plasticity in the domestication of the fox and dog [13, 67, 68]. In fox, for instance, genomic regions that differentiate tame and aggressive animals include *GABBR1* and *GABRA3* and other receptor-coding genes. The closely-related *GABRB2* is also associated with anxiety in chickens [69]. Genes identified in the meta-analysis of cattle temperament add support to this body of evidence.

There are some limitations to our findings. One potential caveat could be the effect of residual population stratification on these results which would increase the number of false positives. In order to control for population stratification, models for each cohort included all available covariates: age, contemporary group (year, stud, sex), the GRM fitted as a random effect, as well as genomic control (${{\lambda }}$ gc) correction in the meta-analysis. Any residual population stratification is likely to be very small as the genomic inflation factor (${{\lambda }}$ gc) is very close to 1 (within cohorts 0.937 $\le {{\lambda }}$ gc $\le$ 1.001 and meta-analysis ${{\lambda }}$ gc = 1.025, [see Additional file 1: Table S1]). We adopted this conservative approach to control for larger traces of LD due to small effective population size in cattle [55, 70].

Another potential caveat is the incomplete validation of the variants identified in the meta-analysis. This may be explained by the lower heritability of docility score [7, 50], the fact that it is a different but correlated trait to flight time, and different genetic backgrounds of the validation cohorts (*Bos taurus*) to the breeds included in the meta-analysis (*Bos indicus* and composite *Bos taurus* and *Bos indicus*) (see Additional file 4: Figure S3). It is important to note that the three breeds for which variants were validated, Charolais, Limousin, and Simmental (continental breeds), are genetically and phenotypically more related to each other, than the Angus and Hereford animals (British breeds) [50]. Moreover, there was more power to estimate variant effects in these continental breeds as these cohorts had the largest sample sizes in the validation GWAS (31,049 Charolais, 35,159 Limousin and 8632 Simmental). In addition, the opposite than expected direction of association for Limousin and Simmental cattle may be due to different haplotypes, at the chromosome 21 locus, between these two breeds and Charolais. However, overall our results provide evidence that the genomic regions associated with temperament in the meta-analysis might also be of importance in other breeds.

There was enrichment for associations with bovine temperament for ASD genes, weaker enrichment for SCZ, but a lack of enrichment for NEU and DDD genes. These results could be due to several reasons: inadequate mapping of susceptibility genes from the human GWAS, lack of concordance of traits between humans and cattle, and different age-of-onset between the diseases. With regard to the mapping of susceptibility genes, NEU and SCZ genes are derived mainly from GWAS data, in which a SNP association may map to multiple genes. In contrast, the ASD and DDD susceptibility genes used here were identified through whole exome sequencing studies in proband-parent trios [35, 36, 71] with some evidence that they are causally associated. However, for ASD only about 10 to 15% of cases can be attributed to rare germline mutations and thus, for most cases, its etiology is polygenic [72], showing relatively weak genetic correlations with other psychiatric and personality disorders [73]. Another potential explanation, based on the evidence for distinct developmental profiles in social communication difficulties [74], is the substantially different age-of-onset for ASD when compared to NEU and SCZ. Temperament in beef cattle is measured in young animals that just reached puberty, 10 to 14 months in our cohorts, and thus it is possible that flight time could be better suited to capture the effect of early age-of-onset disorders, such as ASD. Therefore, the combination of susceptibility genes mapping, number of known de novo mutations, and age-of-onset make ASD a unique disorder among those tested.

As noted before, we are not the first to suggest a connection between ASD and animal behavior. Kukekova et al [13] found an overlap between genes involved in aggression, sociability and anxiety in foxes and genes involved in ASD. One of these overlapping genes is *MAGEL2* and is located in the region containing the most significant variant in the meta-analysis (P = 3 × 10^−10^) that explains 2.2% of the additive genetic variance of cattle temperament (Table 1).

## Conclusions

Our analysis of genetic factors that contribute to variation in temperament traits shared across humans and cattle revealed an association of ASD susceptibility genes with cattle temperament, with *GABRB3* and *CUL3* being the most strongly associated genes. Overall, the genes identified in the meta-analysis contribute to neuron development functions and are differentially expressed in human brain and pituitary tissues. These findings provide quantitative molecular evidence that genetic control of temperament traits might be shared across humans and cattle and highlight the potential for future analyses to leverage results between species, exploiting potential species-specific advantages in experimental designs.

## Supplementary information

**Additional file 1: Table S1.** Sample size, number of imputed sequence variants, estimated heritabilities and ${{\lambda }}$ gc for each cohort used in the meta-analysis. **Table S2.** Validation of variants identified in the flight time meta-analysis (115 variants) in five independent docility score GWAS. **Table S3.** Sign test for lead variants from the flight time meta-analysis using docility score GWAS summary statistics in five independent populations. **Table S4.** Bovine orthologous NEU genes (577 genes). **Table S5.** Bovine orthologous SCZ genes (263 genes). **Table S6.** Bovine orthologous ASD genes (63 genes). **Table S7.** Bovine orthologous DDD genes (101 genes). **Table S8.** Significant genes in the set-based association analysis for mapping the meta-analysis lead variants to genes. **Table S9.** Summary of the candidate genes identified in the meta-analysis of cattle flight time (10 protein-coding genes). **Table S10.** χ2 test of independence for bovine orthologous ASD genes and all UMD3.1 bovine protein-coding genes in brain tissues (cerebellum and caudal lobe combined).

**Additional file 2: Additional methods.** (1) Summary of the methodology for the GWA studies in the validation cohorts from the Irish national breeding program, and (2) Enrichment of ASD genes in bovine brain tissue.

**Additional file 3.** GWAS summary statistics for the cattle flight time meta-analysis. This file contains the GWAS summary statistics for the meta-analysis of cattle flight time. For each of the variants included in the meta-analysis, the following information is provided: MarkerName, Allele1, Allele2, Freq 1, FreqSE, MinFreq, MaxFreq, Effect, StdErr, P-value and Direction.

**Additional file 4: Figure S1.** GWAS meta-analysis results for cattle temperament for SNPs around 100 kb of ASD genes (183,880 SNPs). **Figure S2.** Replication test of ASD genes using bovine RNA-seq data. **Figure S3.** Comparison of the frequency of the reference allele (A1) in the discovery and validation cohorts for the estimated lead variants (3:52892109 and 21:1058688) in the meta-analysis of cattle flight time.

## Data Availability

GWAS summary statistics for the cattle flight time meta-analysis are available in Additional file 3. Gene names, IDs, and locations for bovine orthologous genes implicated in NEU, SCZ, ASD and DDD are in (see Additional file 1: Tables S4–S7). The phenotypic and genotypic data cannot be shared because they are owned by research and commercial breeding programs.

## References

[1] Wray NR, Ripke S, Mattheisen M, Trzaskowski M, Byrne EM, Abdellaoui A (2018). Genome-wide association analyses identify 44 risk variants and refine the genetic architecture of major depression. Nat Genet.

[2] Okbay A, Baselmans BM, De Neve JE, Turley P, Nivard MG, Fontana MA (2016). Genetic variants associated with subjective well-being, depressive symptoms, and neuroticism identified through genome-wide analyses. Nat Genet.

[3] Bipolar Disorder and Schizophrenia Working Group of the Psychiatric Genomics Consortium (2018). Genomic dissection of bipolar disorder and schizophrenia, including 28 subphenotypes. Cell.

[4] Bulik-Sullivan B, Finucane HK, Anttila V, Gusev A, Day FR, Loh PR (2015). An atlas of genetic correlations across human diseases and traits. Nat Genet.

[5] Lo MT, Hinds DA, Tung JY, Franz C, Fan CC, Wang Y (2017). Genome-wide analyses for personality traits identify six genomic loci and show correlations with psychiatric disorders. Nat Genet.

[6] Grove J, Ripke S, Als TD, Mattheisen M, Walters RK, Won H (2019). Identification of common genetic risk variants for autism spectrum disorder. Nat Genet.

[7] Haskell MJ, Simm G, Turner SP (2014). Genetic selection for temperament traits in dairy and beef cattle. Front Genet.

[8] Burrow HM (1997). Measurements of temperament and their relationships with performance traits of beef cattle. Anim Breed Abstracts.

[9] Fordyce G, Goddard ME, Seifert GW (1982). The measurement of temperament in cattle and the effect of experience and genotype. Proc Aust Soc Animal Prod.

[10] Voisinet BD, Grandin T, Tatum JD, OConnor SF, Struthers JJ (1997). Feedlot cattle with calm temperaments have higher average daily gains than cattle with excitable temperaments. J Anim Sci.

[11] Cooke RF, Kunkle BE (2014). Interdisciplinary Beef Symposium: Temperament and acclimation to human handling influence growth, health, and reproductive responses in Bos taurus and Bos indicus cattle. J Anim Sci..

[12] Cooke RF, Moriel P, Cappellozza BI, Miranda VFB, Batista LFD, Colombo EA (2018). Effects of temperament on growth, plasma cortisol concentrations and puberty attainment in Nelore beef heifers. Animal..

[13] Kukekova AV, Johnson JL, Xiang X, Feng S, Liu S, Rando HM (2018). Red fox genome assembly identifies genomic regions associated with tame and aggressive behaviours. Nat Ecol Evol.

[14] Riley DG, Gill CA, Boldt CR, Funkhouser RR, Herring AD, Riggs PK (2016). Crossbred Bos indicus steer temperament as yearlings and whole genome association of steer temperament as yearlings and calf temperament post-weaning. J Anim Sci.

[15] Porto-Neto LR, Reverter A, Prayaga KC, Chan EK, Johnston DJ, Hawken RJ (2014). The genetic architecture of climatic adaptation of tropical cattle. PLoS One.

[16] Valente TS, Baldi F, Sant’Anna AC, Albuquerque LG, Paranhos da Costa MJ (2016). Genome-wide association study between single nucleotide polymorphisms and flight speed in Nellore Cattle. PLoS One.

[17] Glenske K, Prinzenberg EM, Brandt H, Gauly M, Erhardt G (2011). A chromosome-wide QTL study on BTA29 affecting temperament traits in German Angus beef cattle and mapping of DRD4. Animal..

[18] Grandin T, Johnson C (2009). Animals in translation: Using the mysteries of autism to decode animal behavior.

[19] Grandin T, Deesing MJ, Press A (2014). Behavioral genetics and animal science. Genetics and the behavior of domestic animals.

[20] Grandin T (2008). Thinking in pictures, expanded edition: my life with autism.

[21] Burrow HM, Seifert GW, Corbet NJ (1988). A new technique for measuring temperament in cattle. Proc Aust Soc Anim Prod.

[22] Cloninger CR, Svrakic DM, Przybeck TR (1993). A psychobiological model of temperament and character. Arch Gen Psychiatry.

[23] Cloninger CR, Przybeck TR, Svrakic DM, Wetzel RD. The Temperament and Character Inventory (TCI): a guide to its development and use. 1994.

[24] Keller MC, Coventry WL, Heath AC, Martin NG (2005). Widespread evidence for non-additive genetic variation in Cloninger’s and Eysenck’s personality dimensions using a twin plus sibling design. Behav Genet.

[25] Verweij KJ, Zietsch BP, Medland SE, Gordon SD, Benyamin B, Nyholt DR (2010). A genome-wide association study of Cloninger’s temperament scales: implications for the evolutionary genetics of personality. Biol Psychol.

[26] Costa PT, McCrae RR (1990). Personality disorders and the five-factor model of personality. J Pers Disord.

[27] Costa PT, Widiger TA. Introduction: personality disorders and the five-factor model of personality. Personality disorders and the five-factor model of personality (2nd ed); 2002. p. 3–14.

[28] Vukasovic T, Bratko D (2015). Heritability of personality: a meta-analysis of behavior genetic studies. Psychol Bull.

[29] Pryce JE, Hayes BJ, Bolormaa S, Goddard ME (2011). Polymorphic regions affecting human height also control stature in cattle. Genetics.

[30] Bouwman AC, Daetwyler HD, Chamberlain AJ, Ponce CH, Sargolzaei M, Schenkel FS (2018). Meta-analysis of genome-wide association studies for cattle stature identifies common genes that regulate body size in mammals. Nat Genet.

[31] Kemper KE, Visscher PM, Goddard ME (2012). Genetic architecture of body size in mammals. Genome Biol.

[32] Qiu X, Martin GB, Blache D (2017). Gene polymorphisms associated with temperament. J Neurogenet.

[33] Nagel M, Jansen PR, Stringer S, Watanabe K, de Leeuw CA, Bryois J (2018). Meta-analysis of genome-wide association studies for neuroticism in 449,484 individuals identifies novel genetic loci and pathways. Nat Genet.

[34] Pardinas AF, Holmans P, Pocklington AJ, Escott-Price V, Ripke S, Carrera N (2018). Common schizophrenia alleles are enriched in mutation-intolerant genes and in regions under strong background selection. Nat Genet.

[35] Sanders Stephan J, He X, Willsey AJ, Ercan-Sencicek AG, Samocha Kaitlin E, Cicek AE (2015). Insights into autism spectrum disorder genomic architecture and biology from 71 risk loci. Neuron.

[36] Deciphering Developmental Disorders Study (2015). Large-scale discovery of novel genetic causes of developmental disorders. Nature.

[37] Hayes BJ, Daetwyler HD (2019). 1000 bull genomes project to map simple and complex genetic traits in cattle: applications and outcomes. Annu Rev Anim Biosci..

[38] Veerkamp RF, Bouwman AC, Schrooten C, Calus MP (2016). Genomic prediction using preselected DNA variants from a GWAS with whole-genome sequence data in Holstein-Friesian cattle. Genet Sel Evol..

[39] Purfield DC, Evans RD, Berry DP (2019). Reaffirmation of known major genes and the identification of novel candidate genes associated with carcass-related metrics based on whole genome sequence within a large multi-breed cattle population. BMC Genomics..

[40] Pausch H, MacLeod IM, Fries R, Emmerling R, Bowman PJ, Daetwyler HD (2017). Evaluation of the accuracy of imputed sequence variant genotypes and their utility for causal variant detection in cattle. Genet Sel Evol..

[41] Sargolzaei M, Chesnais JP, Schenkel FS (2014). A new approach for efficient genotype imputation using information from relatives. BMC Genomics..

[42] Loh P-R, Danecek P, Palamara PF, Fuchsberger C, Reshef YA, Finucane HK (2016). Reference-based phasing using the Haplotype Reference Consortium panel. Nat Genet.

[43] Das S, Forer L, Schonherr S, Sidore C, Locke AE, Kwong A (2016). Next-generation genotype imputation service and methods. Nat Genet.

[44] Daetwyler HD, Capitan A, Pausch H, Stothard P, van Binsbergen R, Brondum RF (2014). Whole-genome sequencing of 234 bulls facilitates mapping of monogenic and complex traits in cattle. Nat Genet.

[45] Purcell S, Neale B, Todd-Brown K, Thomas L, Ferreira MA, Bender D (2007). PLINK: a tool set for whole-genome association and population-based linkage analyses. Am J Hum Genet.

[46] VanRaden PM (2008). Efficient methods to compute genomic predictions. J Dairy Sci.

[47] Yang J, Lee SH, Goddard ME, Visscher PM (2011). GCTA: a tool for genome-wide complex trait analysis. Am J Hum Genet.

[48] Willer CJ, Li Y, Abecasis GR (2010). METAL: fast and efficient meta-analysis of genomewide association scans. Bioinformatics.

[49] R Core Team. R: A Language and Environment for Statistical Computing. Vienna, Austria: R Foundation for Statistical Computing; 2018.

[50] Doyle JL, Berry DP, Walsh SW, Veerkamp RF, Evans RD, Carthy TR (2018). Genetic covariance components within and among linear type traits differ among contrasting beef cattle breeds. J Anim Sci.

[51] Bakshi A, Zhu Z, Vinkhuyzen AA, Hill WD, McRae AF, Visscher PM (2016). Fast set-based association analysis using summary data from GWAS identifies novel gene loci for human complex traits. Sci Rep..

[52] Szklarczyk D, Morris JH, Cook H, Kuhn M, Wyder S, Simonovic M (2017). The STRING database in 2017: quality-controlled protein-protein association networks, made broadly accessible. Nucleic Acids Res.

[53] GTExConsortium (2015). The genotype-tissue expression (GTEx) pilot analysis: multitissue gene regulation in humans. Science.

[54] Watanabe K, Taskesen E, van Bochoven A, Posthuma D (2017). Functional mapping and annotation of genetic associations with FUMA. Nat Commun..

[55] Bovine HapMap C, Gibbs RA, Taylor JF, Van Tassell CP, Barendse W, Eversole KA (2009). Genome-wide survey of SNP variation uncovers the genetic structure of cattle breeds. Science.

[56] Porto-Neto LR, Kijas JW, Reverter A (2014). The extent of linkage disequilibrium in beef cattle breeds using high-density SNP genotypes. Genet Sel Evol.

[57] Chamberlain AJ, Vander Jagt CJ, Hayes BJ, Khansefid M, Marett LC, Millen CA (2015). Extensive variation between tissues in allele specific expression in an outbred mammal. BMC Genomics..

[58] Comuzzie AG, Cole SA, Laston SL, Voruganti VS, Haack K, Gibbs RA (2012). Novel genetic loci identified for the pathophysiology of childhood obesity in the Hispanic population. PLoS One.

[59] Tanaka M, DeLorey TM, Delgado-Escueta AV, Olsen RW (2010). GABRB3, epilepsy, and neurodevelopment. Epilepsia..

[60] Angulo MA, Butler MG, Cataletto ME (2015). Prader-Willi syndrome: a review of clinical, genetic, and endocrine findings. J Endocrinol Invest.

[61] Ehrhart F, Janssen KJM, Coort SL, Evelo CT, Curfs LMG (2019). Prader-Willi syndrome and Angelman syndrome: visualisation of the molecular pathways for two chromosomal disorders. World J Biol Psychiatry..

[62] Kishimoto R, Tamada K, Liu X, Okubo H, Ise S, Ohta H (2015). Model mice for 15q11-13 duplication syndrome exhibit late-onset obesity and altered lipid metabolism. Hum Mol Genet.

[63] Fountain MD, Tao H, Chen CA, Yin J, Schaaf CP (2017). Magel2 knockout mice manifest altered social phenotypes and a deficit in preference for social novelty. Genes Brain Behav..

[64] Igarashi M, Narayanaswami V, Kimonis V, Galassetti PM, Oveisi F, Jung K-M (2017). Dysfunctional oleoylethanolamide signaling in a mouse model of Prader-Willi syndrome. Pharmacol Res.

[65] Utsunomiya YT, Carmo AS, Neves HH, Carvalheiro R, Matos MC, Zavarez LB (2014). Genome-wide mapping of loci explaining variance in scrotal circumference in Nellore cattle. PLoS One.

[66] Hayes BJ, Corbet NJ, Allen JM, Laing AR, Fordyce G, Lyons R (2019). Towards multi-breed genomic evaluations for female fertility of tropical beef cattle. J Anim Sci.

[67] Kukekova AV, Trut LN, Acland GM, Grandin T, Deesing MJ (2014). Chapter 10—genetics of domesticated behavior in dogs and foxes. Genetics and the behavior of domestic animals.

[68] Li Y, Wang GD, Wang MS, Irwin DM, Wu DD, Zhang YP (2014). Domestication of the dog from the wolf was promoted by enhanced excitatory synaptic plasticity: a hypothesis. Genome Biol Evol..

[69] Johnsson M, Williams MJ, Jensen P, Wright D (2016). Genetical genomics of behavior: a novel chicken genomic model for anxiety behavior. Genetics.

[70] Tenesa A, Navarro P, Hayes BJ, Duffy DL, Clarke GM, Goddard ME (2007). Recent human effective population size estimated from linkage disequilibrium. Genome Res.

[71] De Rubeis S, He X, Goldberg AP, Poultney CS, Samocha K, Ercument Cicek A (2014). Synaptic, transcriptional and chromatin genes disrupted in autism. Nature.

[72] Gaugler T, Klei L, Sanders SJ, Bodea CA, Goldberg AP, Lee AB (2014). Most genetic risk for autism resides with common variation. Nat Genet.

[73] Gandal MJ, Haney JR, Parikshak NN, Leppa V, Ramaswami G, Hartl C (2018). Shared molecular neuropathology across major psychiatric disorders parallels polygenic overlap. Science.

[74] St Pourcain B, Robinson EB, Anttila V, Sullivan BB, Maller J, Golding J (2018). ASD and schizophrenia show distinct developmental profiles in common genetic overlap with population-based social communication difficulties. Mol Psychiatry..

