# Supplementary material for: Genetic control of temperament traits across species: association of autism spectrum disorder risk genes with cattle temperament
Source: Genet Sel Evol. 2020 Aug 26;52:51. doi: 10.1186/s12711-020-00569-z (PMC7448488; doi:10.1186/s12711-020-00569-z)
Supplement: Supplementary file 2 — Additional file 2: Additional methods. (1) Summary of the methodology for the GWA studies in the validation cohorts from the Irish national breeding program, and (2) Enrichment of ASD genes in bovine brain tissue. [file 12711_2020_569_MOESM2_ESM.pdf]

Supplementary Methods

1. Summary of the methodology for the GWA studies in the validation cohorts from the Irish national breeding program

Docility is scored subjective in both commercial and seedstock beef herds by trained classifiers contracted to the Irish Cattle Breeding Federation (DOYLE *et al.* 2018). Scoring is carried out on a linear scale from 1 to 10 where 1 represents aggressive and 10 represents docile. Data on docility were available on 3,356 purebred Angus (AA), 31,049 Charolais (CH), 3,004 Hereford (HE), 35,159 Limousin (LM), and 8,632 Simmental (SI) beef cattle scored between the years 2000 and 2016. All animals were scored between 6 and 16 months of age with a recorded sire and dam. Contemporary group was defined as herd-by-scoring date generated separately per breed. Each contemporary group had to have at least five records.

Generation of adjusted phenotypes

Prior to inclusion in the analysis, all beef cattle phenotypes were adjusted within breed in ASREML (GILMOUR *et al.* 2009) using the model:

$$y = HSD + Sex + AM + DP + Animal + \varepsilon$$

where y is the linear type trait, HSD is the fixed effect of herd by scoring date (11,130 levels), Sex is the sex of the animal (male or female), AM is the fixed effect of the age in months of the animal (11 classes from 6 to 16 months), DP is the fixed effect of the parity of the dam (1, 2, 3, 4 and ≥5), animal is the random additive effect of the animal, and $\boldsymbol{\varepsilon}$ is the random residual effect. The adjusted phenotype was the raw phenotype less the fixed effect solutions of HSD, sex, AM, and DP.

Genotype data

Animals were genotyped using either the Bovine Illumina SNP50, the Illumina High Density (HD), the Illumina 3k panel, the Illumina Low Density genotyping panel or a bespoke genotype

panel (IDB) developed in Ireland (MULLEN *et al.* 2013). Each animal had a call rate ≥ 90% and only autosomal SNPs, SNPs with a known chromosome position on UMD 3.1, and SNPs with a call rate ≥ 90% within panel were retained for imputation.

All genotyped animals were first imputed to HD using FImpute2 (SARGOLZAEI *et al.* 2014) and then to whole genome sequence (WGS) using Eagle (version 2.3.2) (LOH *et al.* 2016), Minimac3 (DAS *et al.* 2016a) and run6.0 of the 1000 Bulls Genomes Project (DAETWYLER *et al.* 2014a) as reference population. The average genotype concordance of imputation to WGS, defined as the proportion of correctly called SNPs versus all SNPs using a validation set of 175 Irish animals, was estimated to be 0.98 (PURFIELD *et al.* 2019).

Of the edited dataset, imputed whole genome sequence data existed for 1,444 AA, 6,433 CH, 1,129 HE, 8,745 LM, and 1,698 SI. Quality control edits were imposed on the imputed sequence genotypes within each of the 6 breeds separately; all SNPs with a minor allele frequency (MAF) ≤ 0.002 or that deviated Hardy-Weinberg equilibrium ($p < 1x10^{-6}$) were removed. Further refinement of the dataset of the 23,943 animals with genotype and phenotype information in the present study was undertaken by removing regions of low imputation accuracy, perhaps due to local mis-assemblies or mis-orientated contigs. These regions were identified using an additional dataset of 147,309 verified parent-progeny relationships as described by PURFIELD *et al.* (2019). Following all SNP edits, 15,531,563, 16,110,951, 15,119,634, 15,336,651, 16,969,734 autosomal SNPs remained for analysis in the AA, CH, HE, LM, and SI populations, respectively.

Association analyses

The association analyses were performed within each breed separately using a linear mixed model in GCTA (YANG *et al.* 2011). Autosomal SNPs from the original HD density panel (i.e., 734,159 SNPs) were used to construct the genomic relationship matrix (GRM). The model used for the within-breed analysis was

$$\mathbf{y} = \boldsymbol{\mu} + \mathbf{x}\mathbf{b} + \mathbf{g} + \boldsymbol{\varepsilon}$$

where **y** is a vector of preadjusted phenotypes, **μ** is the overall mean, **x** is the vector of imputed genotypes, **b** is the additive fixed effect of the candidate SNP to be tested for association, **g** ~ N(0,$\boldsymbol{G}\sigma_g^2$) is the vector of additive genetic effects, where **G** is the genomic relationship matrix calculated from the HD SNP genotypes, and σ_g^2 is the additive genetic variance, and **ε** ~ N(0,$\mathbf{I}\sigma_{\boldsymbol{\varepsilon}}^2$) is the vector of random residual effects, and $\sigma_{\boldsymbol{\varepsilon}}^2$ is the residual variance.

2. Enrichment of ASD genes in bovine brain tissue

We used RNA-seq data from several cattle tissues to investigate tissue specificity of expression of ASD genes in cattle. A bovine gene expression atlas (CHAMBERLAIN *et al.* 2015) contains differential expression (DE) for 18 cattle tissues including brain, white blood, mammary and several others from one lactating Holstein dairy cow (3 technical replicates for each tissue type). For each tissue, we used the number of DE genes in bovine orthologous ASD genes (already mapped for enrichment in cattle temperament) and the overall number of DE genes to calculate log odds-ratio of up and down-regulation. We found that ASD genes were significantly more likely to be expressed in cerebellum and caudal lobe, exhibiting a positive log fold change in expression, when compared to all protein-coding bovine genes (Figure S2). We also combined both brain tissues to run a $\chi 2$ test of independence and found that ASD genes are not a random sample of all bovine protein-coding genes as they displayed higher up-regulation levels (P=7.2 x 10^{-5} in Table S10).

References

Chamberlain, A. J., C. J. Vander Jagt, B. J. Hayes, M. Khansefid, L. C. Marett *et al.*, 2015 Extensive variation between tissues in allele specific expression in an outbred mammal. BMC Genomics 16**:** 993.

Daetwyler, H. D., A. Capitan, H. Pausch, P. Stothard, R. Van Binsbergen *et al.*, 2014a Whole-genome sequencing of 234 bulls facilitates mapping of monogenic and complex traits in cattle. Nature genetics 46**:** 858.

Das, S., L. Forer, S. Schönherr, C. Sidore, A. E. Locke *et al.*, 2016a Next-generation genotype imputation service and methods. Nature genetics 48**:** 1284.

Doyle, J. L., D. P. Berry, S. W. Walsh, R. F. Veerkamp, R. D. Evans *et al.*, 2018 Genetic covariance components within and among linear type traits differ among contrasting beef cattle breeds. J Anim Sci 96**:** 1628-1639.

Gilmour, A. R., B. J. Gogel, B. R. Cullis and R. Thompson, 2009 *ASReml User Guide Release 3.0*. VSN International Ltd, Hemel Hempstead, HP1 1ES, UK.

Loh, P.-R., P. Danecek, P. F. Palamara, C. Fuchsberger, Y. A. Reshef *et al.*, 2016 Reference-based phasing using the Haplotype Reference Consortium panel. Nature genetics 48**:** 1443.

Mullen, M. P., M. C. McClure, J. F. Kearney, S. M. Waters, R. Weld *et al.*, 2013 Development of a custom SNP chip for dairy and beef cattle breeding, parentage and research. Interbull Bulletin.

Purfield, D. C., R. D. Evans and D. P. Berry, 2019 Reaffirmation of known major genes and the identification of novel candidate genes associated with carcass-related metrics based on whole genome sequence within a large multi-breed cattle population. BMC Genomics 20**:** 720.

Sargolzaei, M., J. P. Chesnais and F. S. Schenkel, 2014 A new approach for efficient genotype imputation using information from relatives. BMC Genomics 15.

Yang, J., S. H. Lee, M. E. Goddard and P. M. Visscher, 2011 GCTA: a tool for genome-wide complex trait analysis. Am J Hum Genet 88**:** 76-82.
