# Supplementary material for: Genetic control of temperament traits across species: association of autism spectrum disorder risk genes with cattle temperament
Source: Genet Sel Evol. 2020 Aug 26;52:51. doi: 10.1186/s12711-020-00569-z (PMC7448488; doi:10.1186/s12711-020-00569-z)
Supplement: Supplementary file 4 — Additional file 4: Figure S1. GWAS meta-analysis results for cattle temperament for SNPs around 100 kb of ASD genes (183,880 SNPs). Figure S2. Replication test of ASD genes using bovine RNA-seq data. Figure S3. Comparison of the frequency of the reference allele (A1) in the discovery and validation cohorts for the estimated lead variants (3:52892109 and 21:1058688) in the meta-analysis of cattle flight time. [file 12711_2020_569_MOESM4_ESM.pdf]

Supplementary Figures

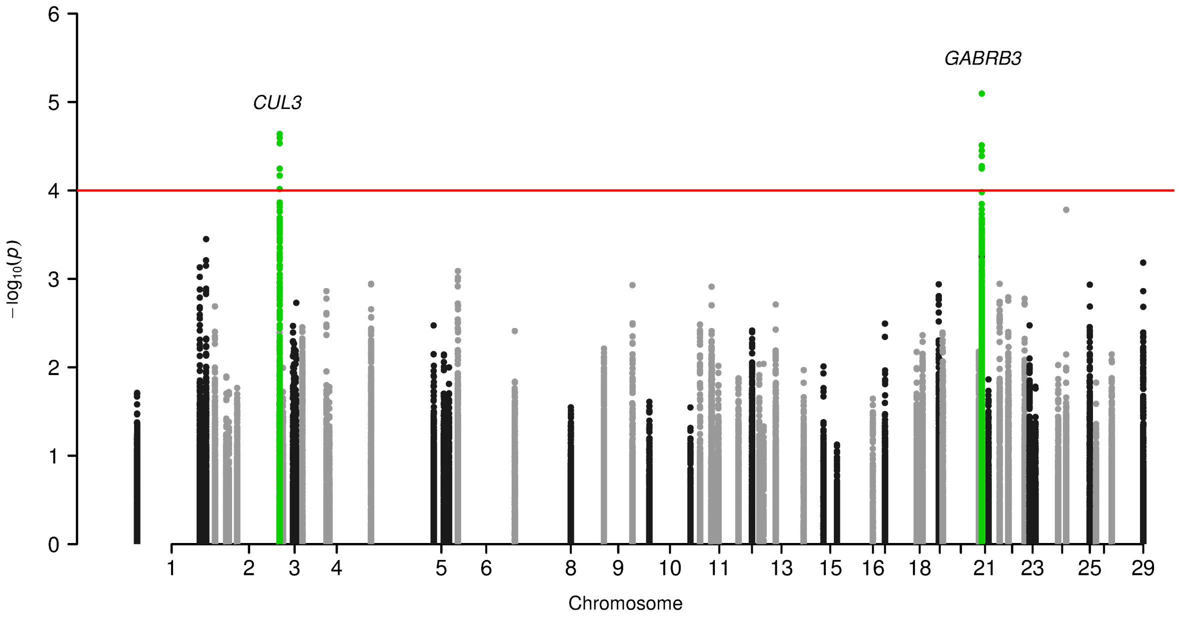

Figure S1. GWAS meta-analysis results for cattle temperament for SNPs around 100Kb of ASD genes (183 880 SNPs). Shown are association statistics [-log_{10}(P)] ordered by genome position.

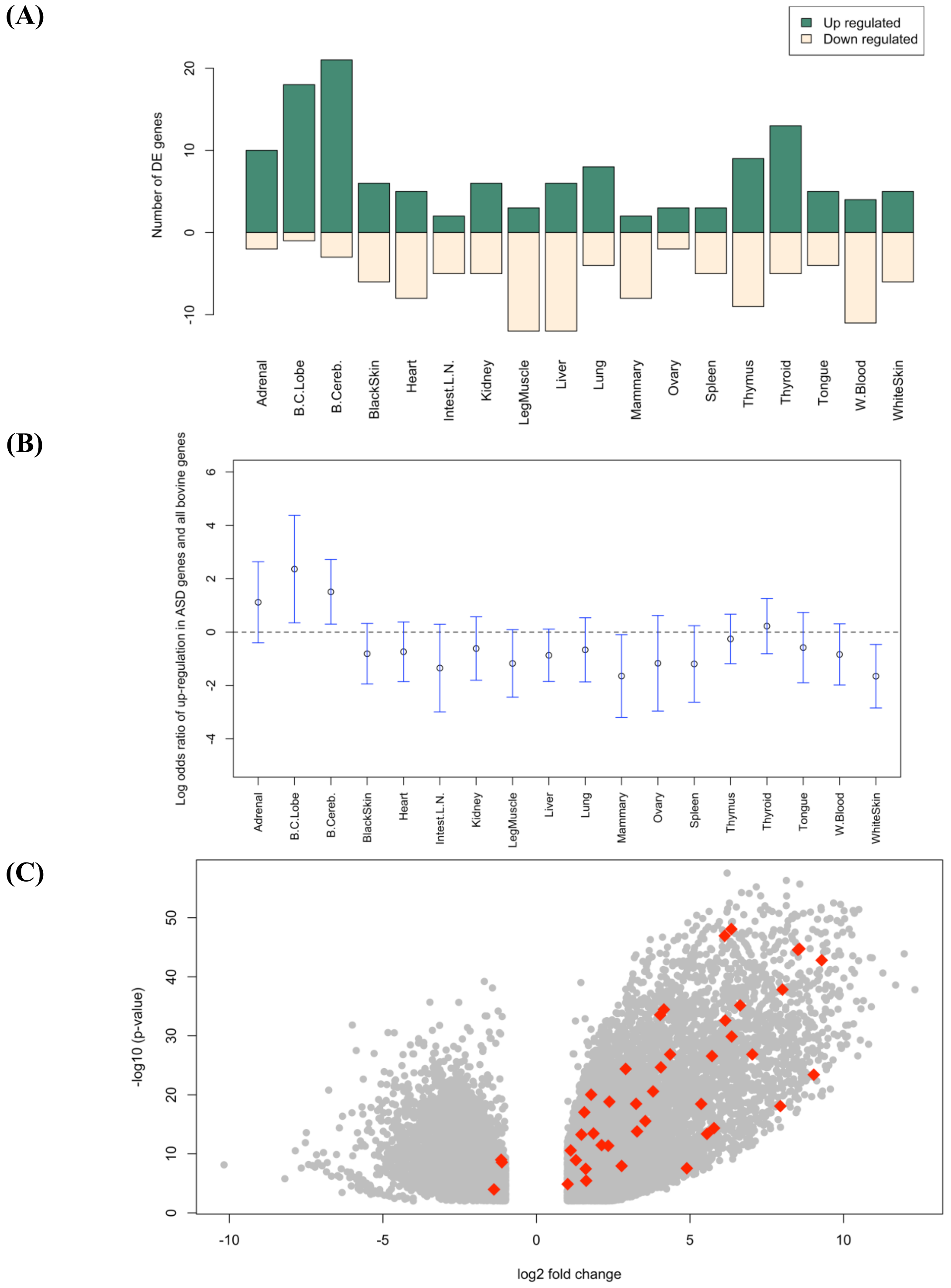

Figure S2. Replication test of ASD genes using bovine RNA-seq data (A) Differentially expressed (DE) ASD genes by tissue. (B) Enrichment in up-regulation of ASD genes by tissue. (C) Comparison of ASD genes (red) and all DE genes (grey) in brain tissues (C. Lobe and Cerebellum combined) using a volcano plot. ASD genes are more up-regulated (positive log2 fold change and smaller p-values) in bovine brain tissues.

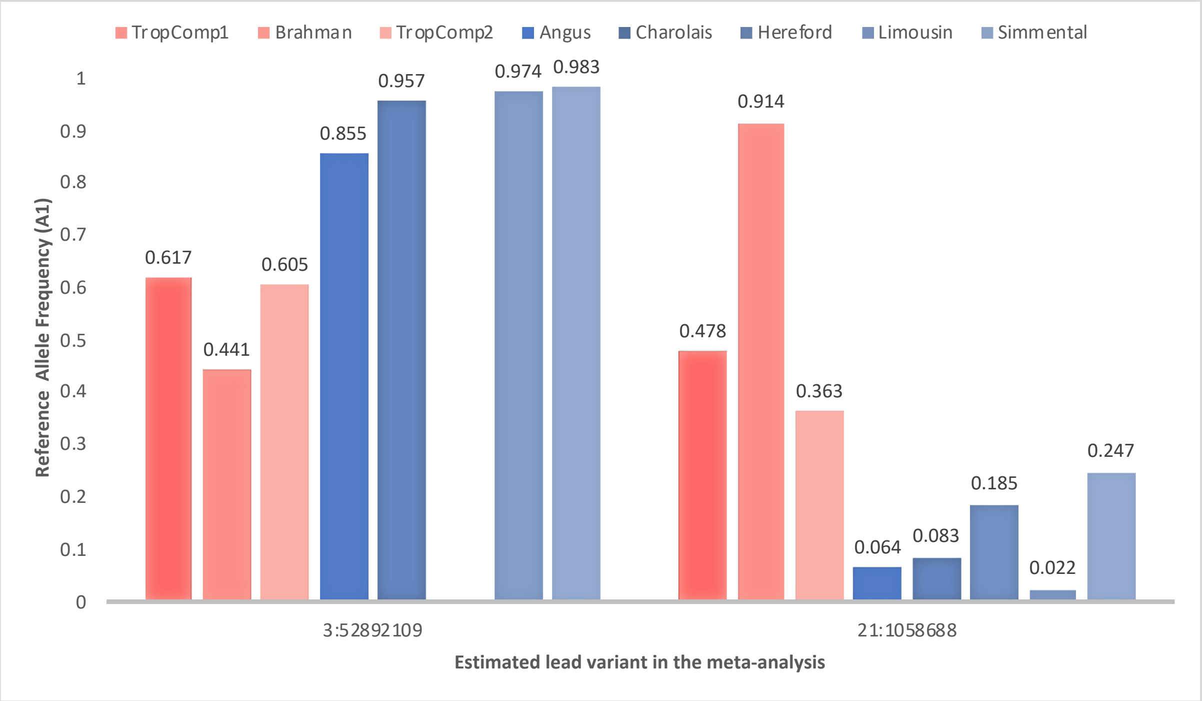

Figure S3. Comparison of the frequency of the reference allele (A1) in the discovery and validation cohorts for the estimated lead variants (3:52892109 and 21:1058688) in the meta-analysis of cattle flight time. Red shaded bars indicate reference allele frequency in discovery cohorts (TropComp1, Brahman, and TropComp2) and blue shaded bars reference allele frequency in validation cohorts (Angus, Charolais, Hereford, Limousin and Simmental). Note that variant 3:52892109 is not polymorphic in the Hereford animals and thus is not shown in the plot.
